# ICA of fMRI Studies: New Approaches and Cutting Edge Applications

**DOI:** 10.3389/fnhum.2013.00724

**Published:** 2013-10-28

**Authors:** Simon Daniel Robinson, Veronika Schöpf

**Affiliations:** ^1^High Field MR Centre, Department of Biomedical Imaging and Image-guided Therapy, Medical University of Vienna, Vienna, Austria; ^2^Department of Biomedical Imaging and Image-guided Therapy, Medical University of Vienna, Vienna, Austria

**Keywords:** independent component analysis, functional magnetic resonance imaging, resting-state networks, schizophrenia, presurgical planning, real-time fMRI, ultra-high field, multiband EPI

Independent component analysis (ICA) is the most commonly used and most diversely applicable exploratory method for the analysis of functional magnetic resonance imaging (fMRI) data. Over the last 10 years it has offered a wealth of insights into brain function during task execution and in the resting state.

Independent component analysis is a blind source separation method that was originally applied to identify technical and physiological artifacts in fMRI, and to allow their removal prior to analysis with model-based approaches. It has matured into a method capable of offering a stand-alone assessment of activation on a sound statistical footing. Recent innovations have taken on the challenges of how components should be combined over subjects to allow group inferences, and how activation identified with ICA might be compared between groups – of patients and controls – for instance. Its reputation having been bolstered by multiple successes in the investigation of resting-state networks, ICA is being applied in other cutting edge uses of fMRI; in multivariate pattern analysis, real-time fMRI, *in utero* studies, with a wide variety of paradigms and stimulus types and with challenging tasks with patients at ultra-high field. These are testament both to ICA's flexibility and its evolving role both in basic neuroscience and clinical applications of fMRI.

This Research Topic has attracted 19 contributions from the most renowned researchers in the field, including the inventor of Fast ICA, Aapo Hyvärinen (Hyvärinen and Ramkumar, 2013), and the authors of the most widely used ICA software for fMRI – Christian Beckmann (FSL's MELODIC) and Vince Calhoun (GIFT). The capacity of ICA to find common patterns of activation in huge cohorts of subjects is demonstrated by the parallel computing approach described by Kalcher et al. (2012) and the use of ICA with cutting edge MR methods are presented by the groups of Stefan Posse [Echo Volume Imaging (Posse et al., 2013)], Markus Barth [EEG-fMRI (Meyer et al., 2013) and Ultra-Fast Generalized Inverse Imaging (Boyacioglu et al., 2013)], and Jorge Jovicich [real-time fMRI (Soldati et al., 2013a,b)].

Two articles in this research topic reflect the continued use of ICA to identify artifacts, using the temporal characteristics of components (Rummel et al., 2013) or both temporal and spatial features (Bhaganagarapu et al., 2013). In addition to using frequency signatures to identify noise, the frequencies of signal fluctuations during rest have been studied using temporal ICA (Boubela et al., 2013) and in ultra-fast generalized imaging (Boyacioglu et al., 2013), while Di et al. (2013) examine the influence of amplitude on resting-state connectivity and Balsters et al. (2013) assess the correlation between BOLD spectral power and working memory performance.

The ICA applications featured in this Research Topic range from clinical resting-state studies with patients suffering from schizophrenia (Manoliu et al., 2013; Sui et al., 2013) and neurological patients performing chin and hand motor tasks (Robinson et al., 2013) to the investigation of processing streams using chemosensory stimuli (Frasnelli et al., 2012). Combined methodological approaches are used to study belief decision making with fMRI and EEG (Douglas et al., 2013), to discriminate schizophrenia using data from fMRI, DTI, and sMRI (Sui et al., 2013), to identify amyotrophic lateral sclerosis diseased brains (Welsh et al., 2013) and to examine the microvascular specificity of the BOLD effect at 3 and 7 T using SWI (Geissler et al., 2013).

We hope this collection of original research articles illustrates the extent to which ICA is becoming an increasingly flexible and potent analysis method – particularly through innovations such as real-time ICA, temporal ICA, and parallel processing implementations – and that the capacity of ICA to isolate the underlying signal sources in fMRI data is being enhanced by multimodal and ultra-fast imaging. These innovations are leading to an increase in the utility of ICA and the richness of information it can provide in both basic research work and clinical applications.
